# Exploring body consciousness of dancers, athletes, and lightly physically active adults

**DOI:** 10.1038/s41598-022-11737-0

**Published:** 2022-05-19

**Authors:** Niia Virtanen, Kaisa Tiippana, Mari Tervaniemi, Hanna Poikonen, Eeva Anttila, Kaisa Kaseva

**Affiliations:** 1 Cognitive Brain Research Unit, Department of Psychology and Logopedics, Faculty of Medicine, University of Helsinki, Helsinki, Finland; 2 Cicero Learning, Faculty of Educational Sciences, University of Helsinki, P.O. Box 9, 00014 Helsinki, Finland; 3 Professorship for Learning Sciences and Higher Education, ETH Zurich, Zurich, Switzerland; 4 Faculty of Arts, University of Helsinki, Helsinki, Finland; 5 Department of Sport and Exercise Medicine, Clinicum, Faculty of Medicine, University of Helsinki, Helsinki, Finland

**Keywords:** Neuroscience, Psychology

## Abstract

Body consciousness is associated with kinetic skills and various aspects of wellbeing. Physical activities have been shown to contribute to the development of body consciousness. Methodological studies are needed in improving the assessment of body consciousness in adults with distinct physical activity backgrounds. This study (1) examined whether dancers, athletes, and lightly physically active individuals differed regarding the level of their body consciousness, and (2) evaluated the usability of different methods in assessing body consciousness. Fifty-seven healthy adults (aged 20–37) were included in the study. Three experimental methods (aperture task, endpoint matching, and posture copying) and two self-report questionnaires (the Private Body Consciousness Scale, PBCS, and the Body Awareness Questionnaire, BAQ) were used in assessing body consciousness. Athletes outperformed the lightly physically active participants in the posture copying task with the aid of vision when copying leg postures. Dancers performed better than the athletes without the aid of vision when their back and upper body were involved, and better than the lightly active participants when copying leg postures. Dancers and athletes had higher self-reported cognitive and perceptual knowledge of their body than lightly physically active participants. To examine the role of different physical activities in developing body consciousness, experimental methods involving the use of the whole body might be most suitable. Subjective measures may provide complementary evidence for experimental testing.

## Introduction

Body consciousness has been shown to be associated with kinetic skills and overall wellbeing^1,2^. It reflects the understanding of what, where, and how one’s body is^3^. The concept covers a range of various kinds of information, including perceptual, semantic, emotional, and cognitive. Specifically, body consciousness has been considered a multisensory phenomenon requiring processing and integration of information from different sensory modalities, including proprioceptive, interoceptive, and exteroceptive information^3^. In the current paper, body consciousness is defined as knowledge of the position and metrics of the body based mainly on visual and proprioceptive information.

In clinical populations, heightened body consciousness has been shown to contribute to sleep and rest patterns, the ability to cope with stressful situations, and overall physiological and psychological balance^4^. Awareness enhancing therapies that help in attending inner sensations can also have physiological and psychological benefits^5^. Furthermore, it has been shown that understanding, caring, and appreciating one’s body contributes to increased psychological wellbeing in healthy individuals^6^. Besides the empirical evidence provided above, speculations that some cultural body ideals may generate personal and social discontent have emerged (e.g.^7^). Thus, dissatisfaction and/ or altered perception of one’s own body and physical qualities may also contribute to the development of mental problems and disorders^8^.

Physical activities are associated with the development of body consciousness (e.g.^1^). Although all activities may contribute to this development, it would be important to understand what types of exercise might be most beneficial and optimal in the formation of people’s consciousness of their physical self. Practising different sports may also require paying attention to focusing on specific body parts. Regarding dancing, body consciousness is an important element both in training and professional practice, as all dancing involves processing information from different sensory modalities^1^. For instance, dancers have been found to have better proprioceptive^9^ and interoceptive^10^ accuracy than people with no background of dancing. Dance practice thus combines introspection, “listening to one’s own body”, with attentive exteroception, “interacting with the environment”^1^, whereas other sports activities (e.g., team sports) may require more focusing on external stimuli^11^. At present, it is still not perfectly clear which specific aspects of information processing might make dancers bodily conscious (i.e., which sensory modalities play the most important role), and whether dancers are more bodily conscious than others with or without a physically active background. Thus, more research on how body consciousness develops and how the development could be measured in healthy adults with distinct physical activity histories is needed^12^. Furthermore, studies on body consciousness are important for specialists interested in developing and optimizing people’s kinetic performance as well as motor skills in a variety of physical activities^11^.

In previous years, a variety of experimental tests and self-report questionnaires have been used in empirical studies on body consciousness. One of the most used experimental methods is the aperture task, in which participants are asked to evaluate whether they could pass through apertures of different widths without turning their shoulders^13^. The task has been used in clinical and general populations^14,15^, and it is regarded as an appropriate method for studying the perception of the size of the body in relation to the environment^13,14^. In the endpoint matching task, locations on a surface are presented to participants and they are asked to indicate the same locations from underneath the surface^9,16^. Dancers rather than non-dancers have been shown to rely more on proprioceptive information when both proprioceptive and visual information about hand position were present^9^.

To gain more information regarding experimental testing, a posture copying task was developed within the present study. Instead of assessing a participant’s ability to estimate the positions of single parts of the body (as e.g., in endpoint matching), posture copying aims to capture participants’ aptitude in positioning their whole body. Participants were instructed to copy postures from photographs by making the same posture with their body with their eyes open and closed. Posture copying required positioning the torso, arms, legs, and head in the same way as shown in the photographs. Performing the task requires visual perception (inspection of the stimuli) and proprioception, an understanding of the body and its movements, and short-term visual and proprioceptive memory.

Numerous self-report questionnaires have also been used in assessing body consciousness, many of these tapping into proprioceptive and interoceptive aspects of body consciousness^5^. With a variety of instruments, the Body Consciousness Questionnaire^17^ and the Body Awareness Questionnaire have demonstrated that they have adequate psychometric properties^5,18^. The Body Consciousness Questionnaire was developed to assess body consciousness in non-affective states^17^, consisting of body competence, public body consciousness, and private body consciousness. The Body Awareness Questionnaire (BAQ) examines an individual’s “beliefs about one’s sensitivity to normal, non-emotive body processes”^18,19^, consisting of a person’s attentiveness to changes in bodily processes, prediction of bodily reactions, awareness of one’s sleep–wake cycle, and prediction of the onset of illness.

Combining objective and subjective methods might be useful in understanding the phenomenon of body consciousness more profoundly. Furthermore, it is important to study whether some specific sensory modalities related to body consciousness have developed differently among people with different physical activity backgrounds, and if specific physical activity histories contribute to heightened consciousness. Such assessments could help professionals, especially those interested in optimizing people’s kinetic performance as well as psychological wellbeing, to choose appropriate methods for assessing body consciousness in future research. Furthermore, this study can assist in developing activities that can potentially strengthen individuals’ consciousness of their physical selves.

The present study assessed dancers, athletes, and lightly physically active participants’ body consciousness using three behavioural methods (aperture task, endpoint matching, and posture copying) and two self-report questionnaires (PBCS and BAQ). Through these assessments, the study aimed to show (1) whether dancers have higher levels of body consciousness than athletes and people with lightly physically active backgrounds, and (2) to examine the applicability of the experimental methods and self-report instruments in assessing body consciousness.

## Methods

Fifty-seven adults participated in the study. The participants were recruited from separate mailing lists for dancers, university students, and employees of various sports centres and Facebook groups for people interested in dance and/or those active in the Finnish street dance community. The sample consisted of healthy adults (i.e., participants who had not been diagnosed with any psychiatric disorder). Participants were allocated to three groups based on the information they reported regarding their physical activity background. The Ethics Review Board in the Humanities and Social and Behavioural Sciences of the University of Helsinki approved the study. All participants gave their written informed consent for publication and participation, and were also naive to the research hypotheses. All methods were designed and applied in accordance with the relevant guidelines and regulations.

The first group consisted of 19 dancers (15 women and 4 men). Inclusion criteria for this group was a finished or ongoing professional education in dance or 5 h or more of dancing per week. Thirteen participants fulfilled both criteria, 2 participants only the former, and 4 participants only the latter. The age of the participants in this group ranged from 24 to 37 years (mean 29.84, SD ± 4.05). Eleven participants had started dancing before the age of 8 and all by the age of 18. The average amount of dance experience was 20.74 years (SD ± 5.03, range 11–23). Ten participants reported contemporary dance as their primary dance style. Other primary dance styles were ballet (4 participants), folk dance (1 participant), carnival samba (1 participant), breakdance (1 participant), show dance (1 participant), and swing (1 participant). All dancers had experience of at least two different dance styles and most had practised a wide array of styles from classical ballet and jazz dance to street, Afro, and Latin dances. Seventeen participants reported they had also practised yoga.

The second group included 19 athletes (13 women and 6 men), who were professional (10 participants) and/or had an education in sports (6 participants) and/or did sports on average for more than five hours weekly (3 participants). Seventeen participants in this group had started doing sports before the age of 11 and all by the age of 16. The age range was 21–36 years (mean 27.26, SD ± 4.13). The mean amount of sports experience was 20.68 years (SD ± 6.02, range 8–31). Participants in the athletes’ group reported currently practising sports. The sports included gym/weight lifting, group exercise, running, combat sports (4 participants), cycling/spinning and swimming. Seven athletes reported that they had practised yoga.

The lightly physically active group consisted of 19 participants (13 women and 6 men) who did not do regular physical exercise. The age range of this group was 20–35 years (mean 26.37, SD ± 4.36). Fifteen of this group’s participants reported they had also practised yoga.

All participants’ self-reported amounts of dance, sports, and musical exercise are presented in Tables 1, 2 and 3. In addition to instructed dance, dancing alone at home or at a club were also considered dancing. Exercise involving music was separated from exercise without music. Musical exercise was defined as exercise done while listening to music (e.g. running to music, group exercise such as aerobics with music, but not dancing). The combined amount of dance and exercise was 5.50–47.50 h/week (mean 17.89, SD ± 11.36) in the dancers’ group, 4.50–19 h/week (mean 9.08, SD ± 3.52) in the athletes’ group, and 0.70–5.50 h/week (mean 2.54, SD ± 1.16) in the physically inactive participants’ group.

**Table 1:** Amount of dance in dancers’, athletes’, and physically inactive participants’ groups (*N* = 57).

Variables	Dancers	Athletes	Inactive subjects
**Dance per week (hours)**			
M (SD)	11.37 (± 7.56)	0.87 (± 0.88)	0.64 (± 0.77)
Range	2.50–30.00	0–3.00	0–3.00
**How often do you dance (*N* of participants)?**			
More often	15		
2–3 times per week	2	2	
Once a week	1	4	1
2–3 times per month		1	3
Once a month		7	8
Once a year		3	6
Not at all		2	2

**Table 2:** Amount of sports in dancers’, athletes’, and physically inactive participants’ groups (*N* = 57).

Variables	Dancers	Athletes	Inactive subjects
**Sports (including exercise with music) per week (hours)**			
M (SD)	6.89 (± 6.66)	8.21 (± 3.50)	1.89 (± 1.21)
Range	1.50–27.50	4.00–17.50	0.70–5.00
**How often do you do sports (including musical exercise)? (*N* of participants)**			
More often	6	15	1
2–3 times per week	10	4	4
Once a week	1		9
2–3 times per month	2		4
Once a month			1
Once a year			
Not at all

**Table 3:** Amount of exercise with music in dancers’, athletes’, and physically inactive participants’ groups (*N* = 57).

Variables	Dancers	Athletes	Inactive subjects
**Musical exercise per week (hours)**			
M (SD)	1.34 (± 2.44)	3.95 (± 3.95)	0.28 (± 0.42)
Range	0–10.00	0–12.50	0.00–1.00
**How many hours of your exercise time is musical exercise (*N* of participants)?**			
More often	1	6	
2–3 times per week	4	5	
Once a week	2	3	3
2–3 times per month	1	1	1
Once a month	2	1	3
Once a year	2		5
Not at all	7	3	7

### Experimental tests

#### Aperture task

The aperture task used in this study was designed according to that developed by Warren and Whang^13^. Apertures of different widths were presented to the participants and they were requested to estimate whether they could walk through the aperture at a normal walking speed without turning their shoulders. The participant stood at a distance of 3 m from two screens (80 × 190 × 3 cm) that were covered with black cloth and placed in front of a light grey wall. The experimenter moved the screens to create apertures of pre-specified widths. The size of the aperture varied from 30 to 85 cm with 5 cm intervals so that each participant was presented with 12 apertures of different widths. Each aperture was presented three times, which resulted in 36 trials. The order of the apertures was randomised. For each trial, participants estimated whether or not they would be able to pass through the aperture without turning their shoulders. The answering time was not limited. Between the trials, the participant held a visual obstruction made of cardboard to prevent them from seeing when changes were made to the width of the aperture.

**Figure 1:**
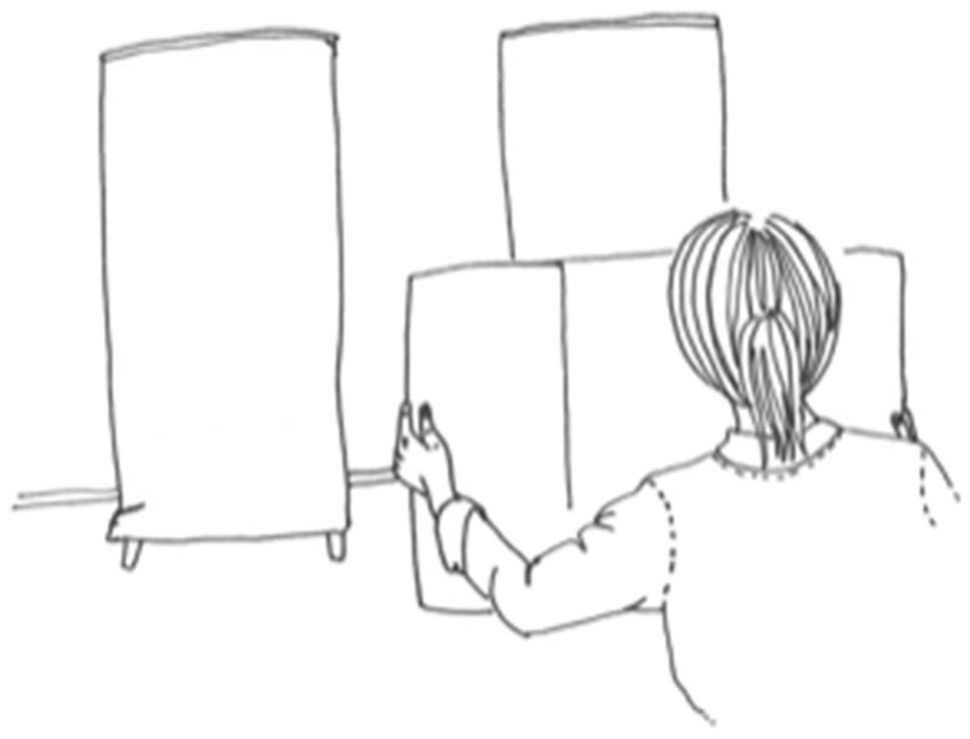
Aperture task.

At the end of the experiment, the width of the widest part of the participant’s body was measured to define the aperture to shoulder width ratio (A/S-ratio). The measurement was carried out by asking participants to stand against one of the screens and slowly pushing the other screen to the other side of the participants’ body so that both screens touched the body (shoulders) but the participants could still move their body through the aperture without turning their shoulders. After positioning the screens, participants were asked to move away from the screens and the aperture left between them was measured. The critical aperture was defined as the widest aperture that participants estimated they could not pass without turning their shoulders in at least two out of three trials. The A/S-ratio was calculated by dividing the critical aperture with the width of the widest part of the participant’s body. The aperture task is illustrated in Fig. 1.

#### Endpoint matching

Three different tasks, proprioceptive, visual, and visuo-proprioceptive, were administered in this order. Randomization of the order was not carried out, because the tasks differed with regard to their difficulty level. Had it been randomized, participants starting with an easier task (visuo-proprioceptive) might have benefitted from the experience gained from it in the more difficult task (proprioceptive). In the first, proprioceptive, task, the participants were blindfolded and the researcher moved the participants’ index finger to one point at a time. The participants showed the location of the point from underneath the table with their other index finger. In the second, visual, task the researcher indicated verbally a number representing a point visible on the table, and participants were asked to first fixate their gaze on the point, and then indicate the location of the point on the lower surface of the table with their index finger. In the third, visuo-proprioceptive, task the researcher indicated verbally a number representing a point, and participants were asked to first fixate their gaze on the point, then place their index finger on the point, and finally place the index finger of their other hand at the same location from underneath the table. In between each matching task in all three conditions participants placed both their hands on their lap or close to their body. Each block was done with both hands. Every other participant started with their right hand while the rest of the participants started with their left hand. Each of the four points was matched five times in every block, which resulted in a total of 120 (5 × 4 × 2 × 3) matching tasks per participant. The participant sat by a table on which was taped a poster (29 × 34 cm) with four randomly arranged points with numbers from 1 to 4. The closest point was 20 cm and the furthest one 36 cm from the participant. The distances between the points varied from 8.5 to 18 cm. Participants sat facing the poster with the poster aligned with their body.

**Figure 2:**
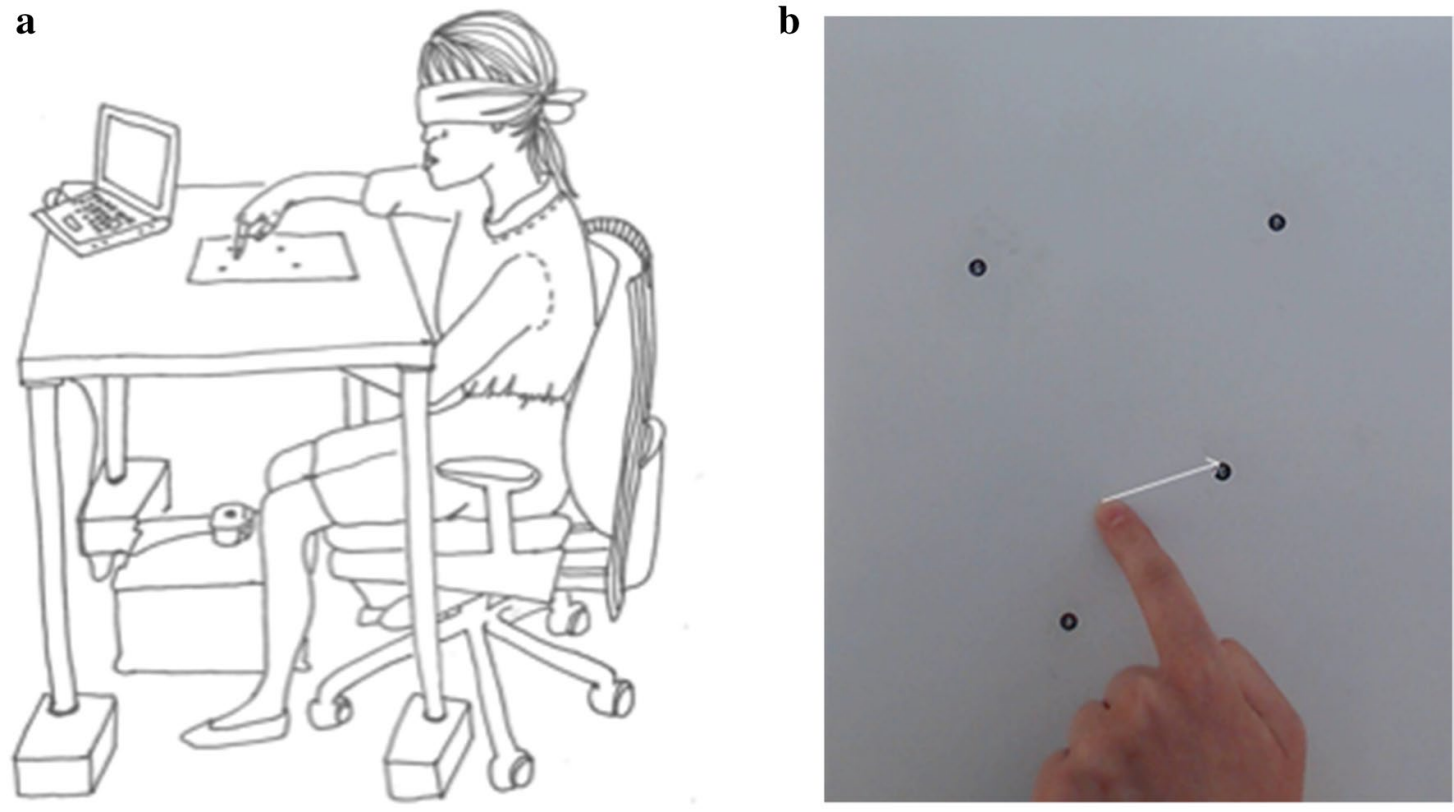
(**a**) Endpoint matching task. (**b**) Photograph of the poster used in endpoint matching task.

A mirror image of the poster was taped on the lower surface of the table so that the locations of the points matched those on top of the table. A camera stand was positioned on the floor underneath the table. A spirit level was used to ensure that the camera was horizontally aligned with the floor and the lower surface of the table. A photograph of the location of the participant’s index finger on the lower surface of the table was taken at each trial. The distances from the finger to the target point (error vectors representing absolute matching acuity) were used in the analysis. The distance from the fingertip to the edge of the target point was calculated in pixels using the measurement tool of a free graphics editor (Gimp, version 2.8). The measurement values were converted into millimetres using the following formula: pixels × 25.4/dpi = mm. The task is illustrated in Fig. 2a and b.

#### Posture copying

**Figure 3:**
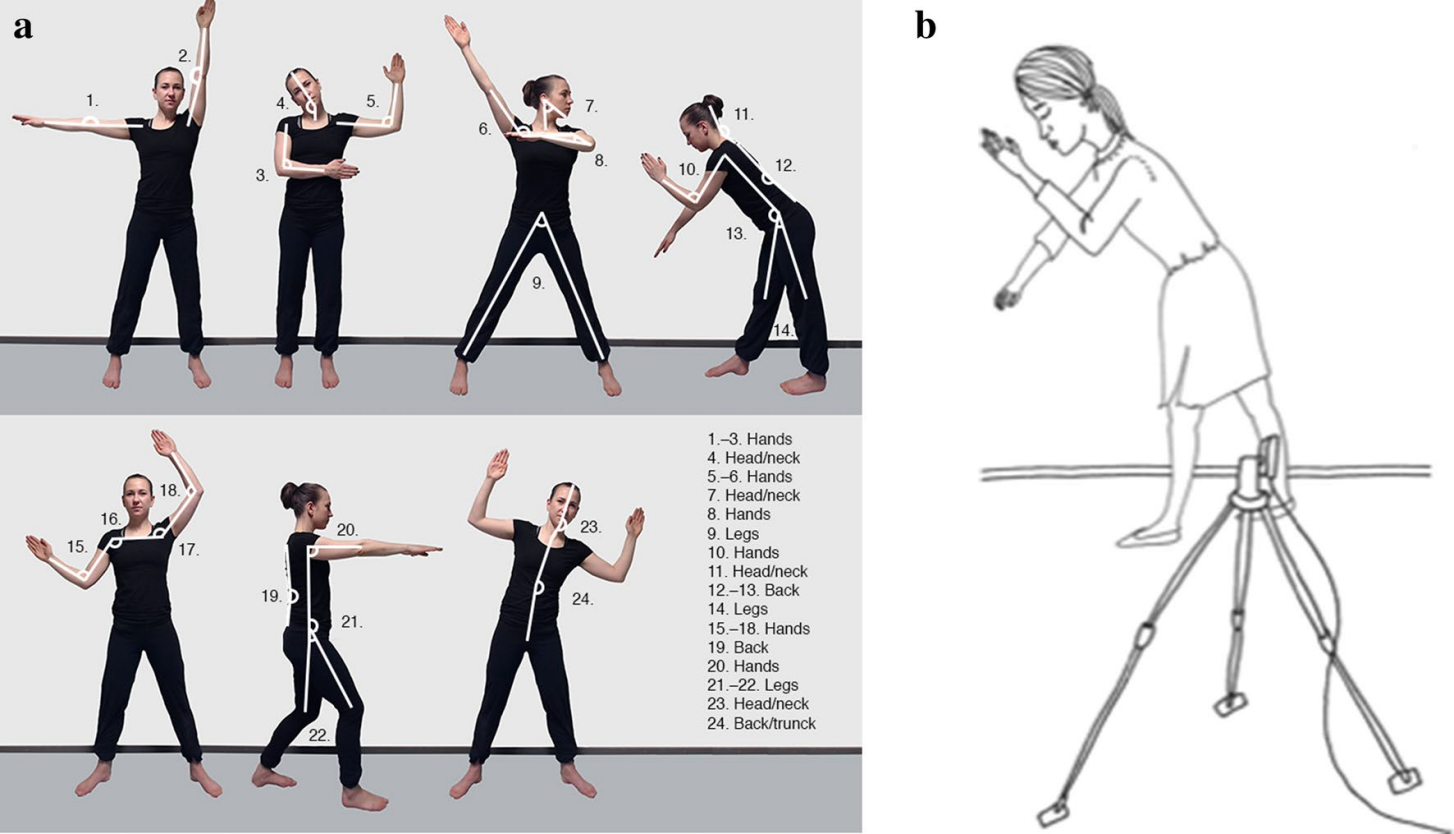
(**a**) Seven two-dimensional posture images for posture copying task. (**b**) Posture copying task.

In the posture copying task, participants were advised to copy postures from photographs by making the same posture with their body. First, the participants were asked to copy the posture with their eyes open. After this, they were advised to return to a specific starting position with their legs slightly apart and their hands close to their body. They were then asked to close their eyes and repeat the previous position they had just copied. Thus, participants copied the same posture with their eyes closed immediately after copying it with their eyes open, and returned to the starting position without breaks. Before starting the actual experiment, participants practised the task with one rehearsal posture and the starting position posture. The stimuli were images of seven two-dimensional postures (Fig. 3a). In addition to one of the authors’ expertise in dance pedagogy, the consultation of a professional dancer was involved in the development of the postures.

Participants stood 3 m from a camera on a tripod. The camera was positioned so that it faced the wall at a 90-degree angle and the horizon of the wall behind the participant was straight. A spirit level was used to ensure the correct position of the camera. Each participant copied seven different postures three times with their eyes open and closed, which resulted in 42 (7 × 3 × 2) copying tasks. Each posture produced by the participant was photographed for later analysis. The postures produced by the participants were analysed by determining the departures from the model images. This was done by calculating the angles formed by the participants’ body parts and comparing the angles in degrees to those in the model images. Thus, the larger the difference in angles, the larger the departure, and thus the poorer the performance. Twenty-four different angles were calculated from the seven model positions using the measurement tool of Photoshop CC. Twelve angles were measured from the hands and shoulders, four from the head and neck, four from the back and upper body, and four from the legs. Eleven variables reflecting the departures were analysed: the total departure between the angles measured from the participant’s postures and the model images, the mean departure separately for eyes open and eyes closed, and body part specific departures for hands, head and neck, back, and legs with both eyes open and closed conditions. The task is illustrated in Fig. 3b.

### Questionnaires

Two self-report questionnaires, the Private Body Consciousness Scale (PBCS) of the Body Consciousness Questionnaire^17^ and the Body Awareness Questionnaire (BAQ)^18^ were administered to all participants. Regarding the original version of the BAQ, the ordering of the questions was mixed and some questions were reversed from negative to positive in order to diminish the potential effect of the participants’ expectations.

The PBCS includes 5 items focusing on physical sensations rated on a 5-point Likert scale ranging from 1 (disagree completely) to 5 (agree completely). The BAQ consists of 18 items measuring attentiveness to changes in bodily processes, prediction of bodily reactions, awareness of one’s sleep–wake cycle, and prediction of the onset of illness. All items in the BAQ are rated on a 7-point Likert scale ranging from 1 (disagree completely) to 7 (agree completely). The sums scores of participants’ responses were calculated for both questionnaires, the PBCS and the BAQ. The maximum value of the PBCS was 25. The maximum value for the BAQ was 126. The reliability of the PBCS was sufficient (Cronbach’s α = 0.66) and was consistent with existing studies (α’s ranging from 0.69 to 0.80)^5,17^. In contrast, the reliability of the BAQ was good (Cronbach’s α = 0.81), which is in alignment with previous findings (α’s ranging from 0.77 to 0.83)^18^. In addition to these two questionnaires, the participants responded to questions capturing information on demographics (e.g., sex), dance, and sports (see Tables 1, 2, 3).

### Statistical analysis

In the endpoint matching task, twelve measurements from ten different participants (two missing values from two different participants and one missing value from eight different participants) were missing or could not be included in the analysis because a participant’s fist obstructed their index finger in the photograph of the endpoint matching location. These values were replaced with the mean value of distance from the participant’s other four matching tasks at the same point in the same block. Since the number of missing values was relatively small (12 missing values from a total of 7200 values), further analysis of missing values was not carried out. The distribution of the visual task was slightly skewed to the right (skewness 1.29), so a logarithmic transformation was applied to ensure normality. Photographs of two separate postures were missing from two participants in the posture copying task. The missing values were replaced with the mean calculated from the participant’s two other images of the respective posture. Because of the relatively small number of missing values (two postures from a total of 2394 postures), no further analysis was carried out. The normality of all variables in all six methods was evaluated visually from histograms and was deemed sufficient unless otherwise stated.

Group differences were analysed with one-way and repeated measures ANOVAs, and Greenhouse–Geisser corrections were used when appropriate. Post hoc-tests’ *p*-values were Bonferroni corrected. Furthermore, eta squared and partial eta squared (η^2^, η_p_^2^) effect size estimates were calculated for the models.

## Results

### Aperture task

The actual physical shoulder widths of participants did not differ between groups [*F*(2,54) = 0.60, *p* = 0.55, η^2^ = 0.02]. The aperture to shoulder width ratios (A/S ratios) measured for the task were 0.94 (± 0.12), 0.95 (± 0.11), and 1.04 (± 0.15) in the dancers’, athletes’, and lightly physically active participants’ groups, respectively. An A/S-ratio of 1.00 implies a perfect estimation of one’s passability through different aperture widths. The groups differed regarding A/S ratios [*F*(2,54) = 3.75, *p* = 0.03, η^2^ = 0.12]. The post hoc tests showed that dancers had marginally lower ratios compared to lightly active participants (mean difference = − 0.10, *p* = 0.048, 95% CI − 0.20 to − 0.00). For details, see Supplemental Table 1.

### Endpoint matching

The groups did not differ from each other in the proprioceptive endpoint matching task [*F*(2,54) = 2.21, *p* = 0.12, η^2^ = 0.8]. The mean error vector in the proprioceptive endpoint matching task was 64 (± 23) mm in the dancers’ group, 55 (± 22) mm in the athletes group, and 74 (± 33) mm in the lightly physically active participants’ group. The groups did not differ from each other in the visual endpoint matching task [*F*(2,54) = 2.46, *p* = 0.10, η^2^ = 0.08]. Corresponding values in the visual task were 59 (± 32) mm, 49 (± 21) mm, and 67 (± 27) mm in the dancers’, athletes’, and lightly active participants’ groups, respectively. The groups did not differ from each other in the visuo-proprioceptive task [*F*(2,54) = 2.55, *p* = 0.09, η^2^ = 0.09]. In the visuo-proprioceptive task the dancers’ averaged error vector was 44 (± 16) mm, the athletes’ 42 (± 17) mm, and the lightly active group’s 54 (± 21) mm. For details, see Supplemental Table 2.

### Posture copying

The departures of the photographs’ models were analysed with a mixed-model ANOVA with Group (3: dancers, athletes, lightly physically active participants) as a between-subjects factor, and Eyes (2: open, closed) and Body part (4: hands, head, back, legs) as repeated measures factors. There was a main effect of Eyes [*F*(1,54) = 24.94, *p* < 0.001, η_p_^2^ = 0.32 ] and Body part [*F*(3,162) = 19.18, *p* < 0.001, η_p_^2^ = 0.26]. The two-way interactions were also significant: Eyes × Body part [*F*(3,162) = 6.99, *p* < 0.001, η_p_^2^ = 0.12], Eyes × Group [*F*(2,162) = 3.61, *p* = 0.03, η_p_^2^ = 0.12], and Body part × Group [*F*(6,162) = 2.57, *p* = 0.03, η_p_^2^ = 0.09]. The 3-way interaction was not significant [*F*(6,162) = 0.77, *p* = 0.64, η_p_^2^ = 0.03]. After conducting the mixed-model ANOVA, significant results were further tested by examining the groups’ performance in each task in eyes open and eyes closed conditions.

#### Tasks performed with eyes open

The groups did not differ in their ability to copy the positions of the hands [*F*(2,54) = 1.79, *p* = 0.18, η^2^ = 0.06]. In the departures calculated from the head and neck, there was a significant difference between groups [*F*(2,54) = 4.01, *p* = 0.02, η^2^ = 0.13]. However, when the Bonferroni-corrected pairwise comparisons were performed, the results did not yield statistical significance (all *p*’s ≥ 0.05).

Regarding the back and upper body, no significant differences were found between the groups [*F*(2,54) = 3.01, *p* = 0.06, η^2^ = 0.10]. With respect to the ability to position one’s legs to correspond to the model pictures, the groups differed from each other [*F*(2,54) = 4.20, *p* = 0.02, η^2^ = 0.14]. The athletes performed better than the lightly physically active participants (mean difference = − 1.81, *p* = 0.03, 95% CI − 3.45 to − 0.17). For details, see Supplemental Table 3.

#### Tasks performed with eyes closed

The groups did not differ in their ability to copy the positions of the hands [*F*(2,54) = 1.25, *p* = 0.29, η^2^ = 0.04]. Regarding the departures calculated from the head and neck, there were no significant differences between the groups [F(2,54) = 3.11, *p* = 0.05, η^2^ = 0.10].

The groups differed in their ability to match the position of the back and upper body with the model postures [*F*(2,54) = 4.53, *p* = 0.02, η^2^ = 0.14]. According to the post hoc tests, dancers performed better than athletes (mean difference = − 3.51, *p* = 0.01, 95% CI − 6.39 to − 0.63). Regarding the ability to position one’s legs to correspond with model pictures, there was a significant difference between the groups [*F*(2,54) = 3.61, *p* = 0.03, η^2^ = 0.12]. Dancers performed better than the lightly active participants (mean difference = − 1.96, *p* = 0.04, 95% CI − 3.82 to − 0.10). For details, see Supplemental Table 4.

### The PBCS and the BAQ

Mean sums in the dancers’, athletes’, and lightly physically active participants’ groups in the PBCS were 19.84 (SD ± 3.04), 20.00 (SD ± 3.18), and 18.53 (SD ± 2.97), respectively. The corresponding values in the BAQ were 98.68 (SD ± 8.76), 95.74 (SD ± 12.14), and 85.42 (SD ± 12.72), respectively. The groups did not differ from each other in the PBCS [*F*(2,54) = 3.15, *p = 0.05,* η^2^ = 0.11].

There was a significant difference between the groups regarding the BAQ [*F*(2,54) = 7.16, *p* = 0.00, η^2^ = 0.21]. Dancers had higher scores in the BAQ compared to lightly active participants (mean difference = 13.26, *p* = 0.00, 95% CI 4.17 to 22.36). The BAQ score was also higher in athletes than in lightly active participants (mean difference = 10.32, *p* = 0.02, 95% CI 1.22 to 19.41). For details, see Supplemental Table 5.

## Discussion

This study assessed the body consciousness of dancers, athletes, and people with no background of regular physical activity using experimental methods and self-report questionnaires. According to our knowledge, the current study is the first in which the dancers’ body consciousness was compared not only to physically lightly active adults, but also to physically active people who do not dance actively. Furthermore, this study is, to our knowledge, the first that provides evidence on different methods’ usability in assessing body consciousness in dancers, athletes and lightly physically active adults. The present work combined evidence from experimental tests and well-validated self-report questionnaires.

In the aperture task, dancers differed marginally from lightly active participants regarding their ability to estimate their ability to pass through different aperture widths. All participants were, however, quite accurate in the task. While there is evidence of eating disorder patients’ relative overestimation in this task^14,15^, this study demonstrated that dancers, athletes and lightly active individuals from the general population appear to have a realistic understanding of the size of their bodies. Furthermore, no group differences were found in the endpoint matching task. This is in contrast with the previous research showing that dancers succeeded better than controls in the proprioceptive version of the task^9^.

Regarding the posture copying task, athletes outperformed the lightly physically active participants with the aid of vision when copying leg postures. Dancers performed better than the athletes without the aid of vision when their back and upper body were involved, and better than the lightly active participants when copying leg postures. Dancers were also marginally better than lightly physically active adults in copying the head and neck postures with eyes closed. Thus, it is possible that dancers are more advanced in perceiving and producing positions involving the whole body than just their hands comparing to other groups. This difference in body consciousness between the groups was found without the aid of vision, which is consistent with previous research in which dancers have been found to have better proprioceptive accuracy as well as a high reliance on proprioception even when visual information is available^9,20^.

Regarding participants’ questionnaire responses, both dancers and athletes differed from lightly active participants regarding the results from the BAQ. Specifically, dancers and athletes had higher self-reported cognitive and perceptual knowledge of their body than lightly physically active participants. The finding is in agreement with previous research indicating that dancers are more confident than controls with regard to their ability to track their own heart rate, considered as a measure of interoceptive accuracy^10^. No differences were found in the PBCS between groups. Similar results regarding this instrument have also been demonstrated in another study on dancers and non-dancers^21^.

In light of the results from the present study, future research on the effects of dance on body consciousness would benefit from employing methods that involve the use of the body as a whole. The posture copying task developed in this study might be suitable for this. Subjective measures, such as the BAQ, might provide complementary evidence for experimental testing. Furthermore, the tested methods may assist in sorting out strategies through which the different kinetic skills needed in a variety of physical activities can be developed.

### Limitations

In the endpoint matching task, the groups may not have differed from each other due to the fact that the method requires only the utilization of hands. All of us, regardless of our dance or exercise experience, frequently use our hands. Dance and many physical activities, however, usually require moving the body as a whole. Furthermore, the different arrangement of target points in the current and previous studies may have affected the findings since the size of the matching error is dependent on the location of the target points^9^.

Another limitation of the current study may be the similarities between participant groups. Dancers often engage in various types of exercise and, vice versa, athletes often also practise dance, which was the case in this study as well. In all groups, there were some participants who had some experience of yoga. Future research would benefit from larger sample sizes, an equal number of male and female participants, as well as more rigorous participant selection with regard to the amount and types of dance and exercise practised by participants, particularly yoga.

Third, the present study is a cross-sectional one, and the results may not be solely attributable to dancing or practising sports. For example, it might also be that sensitivity to the messages of one’s body can lead individuals to participate in dancing or other sports activities. One possibility for evaluating the actual influence of dancing or sports would be a longitudinal study in which a group of individuals receive a dance or sports intervention and will thereafter be compared to a control group consisting of physically inactive individuals.

Neuroscientific studies have also shown that dance training through actual movement shapes the brain processes more than merely watching dance or listening to music^22^. There is, moreover, some evidence of the link between visual experience and emphatic abilities to motor expressions^23^. More studies are still called for to assess the level of visual experience needed for direct motor matching responses, and their stability over time^23^. It would also be important to evaluate to what degree the levels of body consciousness might mediate the association between practice and performance within a variety of physical activities and dance.

## Conclusions

Athletes outperformed the lightly physically active participants in the posture copying task with the aid of vision when copying leg postures. Dancers performed better than the athletes without the aid of vision when their back and upper body were involved, and better than the lightly active participants when copying leg postures. Dancers and athletes also had higher self-reported cognitive and perceptual knowledge of their body than lightly physically active participants. To examine the role of different types of physical activities in the body consciousness, methods involving the use of the whole body might be most suitable. Subjective measures, such as the BAQ, might provide complementary evidence for experimental testing.

## Supplementary Information

Supplementary Tables.

## Data Availability

The data collected during the current study is not publicly available, as the participants were not informed of the possibility that their data would be openly available prior to data acquisition. However, the data can be made available for research use upon a reasonable request addressed to the authors.
