# Supplementary material for: Exploring body consciousness of dancers, athletes, and lightly physically active adults
Source: Sci Rep. 2022 May 19;12:8353. doi: 10.1038/s41598-022-11737-0 (PMC9120170; doi:10.1038/s41598-022-11737-0)
Supplement: Supplementary file 1 — Supplementary Tables. [file 41598_2022_11737_MOESM1_ESM.docx]

**Supplemental Table 1** Bonferroni-corrected pairwise comparisons between the groups (*N*=57) in the

Aperture task.

| Variable |  | Mean difference | | *p* | | 95%CI  (lower) | | 95%CI (upper) |
| --- | --- | --- | --- | --- | --- | --- | --- | --- |
| **Shoulder widths** |  |  |  | |  | | |  |
|  |  |  |  | |  | | |  |
| Dancers | Athletes  Light-activity group | -1.23  -0.73 | 0.85  0.99 | | -4.02  -3.52 | | 1.56  2.06 | |
| Athletes | Dancers  Light-activity group | 1.23  0.50 | 0.85  0.99 | | -1.56  -2.29 | | 4.02  3.29 | |
| Light-activity group | Dancers  Athletes | 0.73  -0.50 | 0.99  0.99 | | -2.06  -3.29 | | 3.52  2.29 | |
|  |  |  |  | |  | |  | |
| **A/S ratio** |  |  |  | |  | |  | |
| Dancers | Athletes  Light-activity group | -0.01  -0.10 | 0.99  0.048 | | -0.11  -0.20 | | 0.09  -0.00 | |
| Athletes | Dancers  Light activity group | -0.01  -0.09 | 0.99  0.09 | | -0.09  -0.19 | | 0.11  0.01 | |
| Light-activity group | Dancers  Athletes | 0.10  0.09 | 0.048  0.09 | | 0.00  -0.01 | | 0.20  0.19 | |

**Supplemental Table 2** Bonferroni-corrected pairwise comparisons between the groups (*N*=57) in the

Endpoint matching task.

| Variable |  | Mean difference | | *p* | | 95%CI (lower) | | 95%CI  (upper) |
| --- | --- | --- | --- | --- | --- | --- | --- | --- |
|  |  |  | |  | |  | |  |
| **Proprioceptive task** |  |  | |  | |  | |  |
| Dancers | Athletes  Light-activity group | 10.01  -8.39 | 0.78  0.99 | | -11.65  -30.05 | | 31.66  13.27 | |
| Athletes | Dancers  Light-activity group | -10.01  -18.40 | 0.78  0.12 | | -31.66  -40.05 | | 11.65  3.26 | |
| Light-activity group | Dancers  Athletes | 8.39  18.40 | 0.99  0.12 | | -13.27  -3.26 | | 30.05  40.05 | |
|  |  |  |  | | |  | |  |
| **Visual task** |  |  |  | | |  | |  |
| Dancers | Athletes  Light-activity group | 8.59  -10.39 | 0.96  0.69 | | | -12.57  -31.55 | 29.76  10.78 | |
| Athletes | Dancers  Light-activity group | -8.59  -18.98 | 0.96  0.09 | | | -29.76  -40.14 | 12.57  2.18 | |
| Light-activity group | Dancers  Athletes | 10.39  18.98 | | 0.69  0.09 | | -10.78  -2.18 | 31.55  40.14 | |
|  |  |  | |  | |  |  | |
| **Visuo-propriopceptive task** |  |  | |  | |  |  | |
| Dancers | Athletes  Light-activity group | 2.52  -10.18 | | 0.99  0.28 | | -12.02  -24.90 | 17.25  4.54 | |
| Athletes | Dancers  Light activity group | -2.52  -12.70 | | 0.99  0.11 | | -17.25  -27.43 | 12.20  2.02 | |
| Light-activity group | Dancers  Athletes | 10.18  12.70 | | 0.28  0.11 | | -4.54  -2.02 | 24.90  27.43 | |

**Supplemental Table 3** Bonferroni-corrected pairwise comparisons between the groups (*N*=57) in the

Posture copying task eyes open.

| Variables |  | Mean difference | *p* | 95%CI  (lower) | | 95%CI (upper) |
| --- | --- | --- | --- | --- | --- | --- |
| **Hands** |  |  |  |  |  | |
|  |  |  |  |  |  | |
| Dancers | Athletes  Light-activity group | -1.29  -0.48 | 0.20  0.99 | -3.00  -2.19 | 0.41  1.22 | |
| Athletes | Dancers  Light-activity group | 1.29  0.81 | 0.20  0.74 | -0.41  -0.90 | 3.00  2.51 | |
| Light-activity group | Dancers  Athletes | 0.48  -0.81 | 0.99  0.74 | -1.22  -2.51 | 2.19  0.90 | |
|  |  |  |  |  |  | |
| **Head and neck** |  |  |  |  |  | |
| Dancers | Athletes  Light-activity group | -0.01  -2.65 | 0.99  0.05 | -2.68  -5.32 | 2.66  0.02 | |
| Athletes | Dancers  Light activity group | 0.01  -2.64 | 0.99  0.05 | -2.66  -5.31 | 2.68  0.03 | |
| Light-activity group | Dancers  Athletes | 2.65  2.64 | 0.05  0.05 | -0.02  -0.03 | 5.32  5.31 | |
| **Back and upper body** |  |  |  |  |  | |
|  |  |  |  |  |  | |
| Dancers | Athletes  Light-activity group | -2.44  -0.66 | 0.06  0.99 | -4.98  -3.20 | 0.10  1.88 | |
| Athletes | Dancers  Light activity group | 2.44  1.78 | 0.06  0.27 | -0.10  -0.76 | 4.98  4.31 | |
| Light-activity group | Dancers  Athletes | 0.66  -1.78 | 0.99  0.27 | -1.88  -4.31 | 3.20  0.76 | |
| **Legs** |  |  |  |  |  | |
| Dancers | Athletes  Light-activity group | 0.34  -1.47 | 0.99  0.09 | -1.30  -3.12 | 1.98  0.17 | |
| Athletes | Dancers  Light activity group | -0.34  -1.81 | 0.99  0.03 | -1.98  -3.45 | 1.30  -0.17 | |
| Light-activity group | Dancers  Athletes | 1.47  1.81 | 0.09  0.03 | -0.17  0.17 | 3.12  3.45 | |

**Supplemental Table 4** Bonferroni-corrected pairwise comparisons between the groups (*N*=57) in the

Posture copying task eyes closed.

| Variables |  | Mean difference | | *p* | | 95%CI (lower) | | 95%CI (upper) |
| --- | --- | --- | --- | --- | --- | --- | --- | --- |
|  |  |  |  | |  | |  | |
| **Hands** |  |  |  | |  | |  | |
| Dancers | Athletes  Light-activity group | -1.13  -0.71 | 0.37  0.98 | | -2.91  -2.50 | | 0.65  1.07 | |
| Athletes | Dancers  Light-activity group | 1.13  0.41 | 0.37  0.99 | | -0.65  -1.37 | | 2.91  2.19 | |
| Light-activity group | Dancers  Athletes | 0.71  -0.41 | 0.98  0.99 | | -1.07  -2.19 | | 2.50  1.37 | |
|  |  |  |  | |  | |  | |
| **Head and neck** |  |  |  | |  | |  | |
| Dancers | Athletes  Light-activity group | -0.75  -2.72 | 0.99  0.06 | | -3.54  -5.51 | | 2.03  0.06 | |
| Athletes | Dancers  Light activity group | 0.75  -2.72 | 0.99  0.26 | | -2.03  -4.76 | | 3.54  0.82 | |
| Light-activity group | Dancers  Athletes | 2.72  1.97 | 0.06  0.26 | | -0.06  -0.82 | | 5.51  4.76 | |
| **Back and upper body** |  |  |  | |  | |  | |
|  |  |  |  | |  | |  | |
| Dancers | Athletes  Light-activity group | -3.51  -1.59 | 0.01  0.53 | | -6.39  -4.48 | | -0.63  1.29 | |
| Athletes | Dancers  Light-activity group | 3.51  1.92 | 0.01  0.32 | | 0.63  -0.97 | | 6.39  4.48 | |
| Light-activity group | Dancers  Athletes | 1.59  -1.92 | 0.53  0.32 | | -1.29  -4.80 | | 4.48  0.97 | |
| **Legs** |  |  |  | |  | |  | |
| Dancers | Athletes  Light-activity group | -0.54  -1.96 | 0.99  0.04 | | -2.40  -3.82 | | 1.32  -0.10 | |
| Athletes | Dancers  Light activity group | 0.54  -1.42 | 0.99  0.20 | | -1.32  -3.28 | | 2.40  0.44 | |
| Light-activity group | Dancers  Athletes | 1.96  1.42 | 0.04  0.20 | | 0.10  -0.44 | | 3.82  3.28 | |

**Supplemental Table 5** Bonferroni-corrected pairwise comparisons between the groups (*N*=57) in the

PBCS and BAQ.

| Variables |  | Mean difference | | p | | 95%CI (lower) | | 95%CI (upper) |
| --- | --- | --- | --- | --- | --- | --- | --- | --- |
| **PBCS** |  |  | |  | |  | |  |
|  |  |  | |  | |  | |  |
| Dancers | Athletes  Light-activity group | -0.21  4.53 | 0.99  0.12 | | -5.48  -0.74 | | 5.06  9.79 | |
| Athletes | Dancers  Light-activity group | 0.21  4.74 | 0.99  0.09 | | -5.06  -0.53 | | 5.48  10.00 | |
| Light-activity group | Dancers  Athletes | -4.53  -4.74 | 0.12  0.09 | | -9.79  -10.00 | | 0.74  0.53 | |
|  |  |  |  | |  | |  | |
| **BAQ** |  |  |  | |  | |  | |
| Dancers | Athletes  Light-activity group | 2.95  13.26 | 0.99  0.00 | | -6.15  4.17 | | 12.04  22.36 | |
| Athletes | Dancers  Light-activity group | -2.95  10.32 | 0.99  0.02 | | -12.04  1.22 | | 6.15  19.41 | |
| Light-activity group | Dancers  Athletes | -13.26  10.32 | 0.00  0.02 | | -22.36  -19.41 | | -4.17  -1.22 | |
